# Differential Associations of Deprivation and Threat With Cognitive Control and Fear Conditioning in Early Childhood

**DOI:** 10.3389/fnbeh.2019.00080

**Published:** 2019-05-08

**Authors:** Laura Machlin, Adam Bryant Miller, Jenna Snyder, Katie A. McLaughlin, Margaret A. Sheridan

**Affiliations:** ^1^Department of Psychology and Neuroscience, The University of North Carolina at Chapel Hill, Chapel Hill, NC, United States; ^2^Cooper Medical School of Rowan University, Camden, NJ, United States; ^3^Department of Psychology, Harvard University, Cambridge, MA, United States

**Keywords:** deprivation, threat, cognitive control, fear conditioning, physiological reactivity

## Abstract

Early-life adversity (ELA) is strongly associated with risk for psychopathology. Within adversity, deprivation, and threat may lead to psychopathology through different intermediary pathways. Specifically, deprivation, defined as the absence of expected cognitive and social inputs, is associated with lower performance on complex cognitive tasks whereas threatening experiences, defined as the presence of experiences that reflect harm to the child, are associated with atypical fear learning and emotional processes. However, distinct associations of deprivation and threat on behavioral outcomes have not been examined in early childhood. The present study examines how deprivation and threat are associated with cognitive and emotional outcomes in early childhood. Children 4–7 years old completed behavioral tasks assessing cognitive control (*N* = 58) and fear conditioning (*N* = 45); deprivation and threat were assessed using child interview and parent questionnaires. Regression analyses were performed including deprivation and threat scores and controls for age, gender, and IQ. Because this is the first time these variables have been examined in early childhood, interactions with age were also examined. Deprivation, but not threat was associated with worse performance on the cognitive control task. Threat, but not deprivation interacted with age to predict fear learning. Young children who experienced high levels of threat showed evidence of fear learning measured by differential skin conductance response even at the earliest age measured. In contrast, for children not exposed to threat, fear learning emerged only in older ages. Children who experienced higher levels of threat also showed blunted reactivity measured by amplitude of skin conductance response to the reinforced stimuli regardless of age. Results suggest differential influences of deprivation and threat on cognitive and emotional outcomes even in early childhood. Future work should examine the neural mechanisms underlying these behavioral changes and link changes with increased risk for negative outcomes associated with adversity exposure, such as psychopathology.

## Introduction

Early-life adversity (ELA) impacts over half of the US population and is strongly associated with risk for psychopathology (Green et al., 2010; McLaughlin et al., 2012; McLaughlin, 2016). Children who experience early adversity are at higher risk for mood disorders, anxiety disorders, substance use disorders, and disruptive behavior disorders with similar strengths of association across mental disorders (Green et al., 2010; Kessler et al., 2010; McLaughlin et al., 2012). Overall, early adversity accounts for nearly one-third of all psychiatric disorder onsets in adolescence and up to 45% of childhood-onset disorders (Green et al., 2010; McLaughlin et al., 2012). It is vital to better understand the developmental processes through which early adversity impacts psychopathology and behavior.

**Figure 1:**
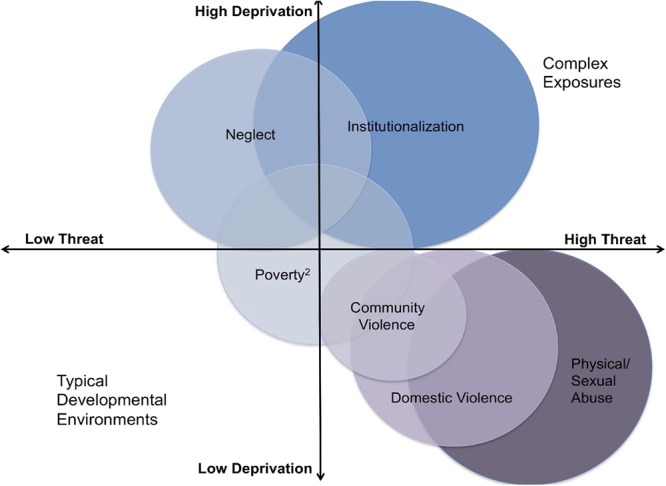
Image of the proposed dimensions of experience of deprivation and threat that may occur in isolation or co-occur. Complex exposures refers to experiences involving both deprivation and threat (Sheridan and McLaughlin, 2014).

Some prior research has focused on associations of single types of early adversity or the number of adverse experiences (i.e., cumulative risk) with psychopathology (Dube et al., 2003; Anda et al., 2005). However, our group has articulated an alternative approach, the dimensional model of adversity and psychopathology (DMAP). In this model, we hypothesize that specific developmental mechanisms link different types of adversity with mental health outcomes (McLaughlin et al., 2014; Sheridan and McLaughlin, 2014, 2016; McLaughlin and Sheridan, 2016). In contrast to cumulative risk models, we differentiate between the impact of deprivation (the absence of expected cognitive and social learning experiences) and threat (experiences reflecting harm or threat of harm to the child) on cognitive and emotional function (Figure 1).

### Deprivation

In the DMAP, deprivation is conceptualized as the absence of or lack of complexity in expected cognitive and social inputs in infancy and early childhood. At the extreme end of deprivation are children who have experienced institutionalization or neglect. However, children who are raised in more typical families with low socioeconomic status (SES) are at higher risk for the absence of expected inputs, including less exposure to language and cognitive stimulation in the home (Hart and Risley, 1995; Bradley and Corwyn, 2002). We expect decreased early cognitive and social stimulation to lead to reductions in complex cognitive abilities later in development including deficits in cognitive control and linguistic ability.

Substantial work from animal models has established a framework for how deprivation may shape neural structure and function. Rodents in deprived environments show decreases in brain volume, cortical weight, cortical thickness, synapses, and dendritic branching (Diamond et al., 1972; Uylings et al., 1978; Würbel, 2001). In adulthood, these effects are partially reversible through exposure to cognitively stimulating environments following the period of deprivation (Diamond et al., 1972).

Institutionalization is a profound experience of deprivation, including less interaction with caregivers, disturbances in attachment, fewer opportunities for social stimulation, and less exposure to cognitive stimuli (Smyke et al., 2002; Zeanah et al., 2005; Nelson et al., 2007; Tottenham, 2012b). Institutionalization is associated with decreases in IQ, language delays, and worse executive functioning (Bos et al., 2009; Loman et al., 2009; Sheridan et al., 2010; Windsor et al., 2011, 2013). Low SES, a risk factor associated with deprivation, is typically comprised of parental education, parental occupation, and family income (Bradley and Corwyn, 2002). Children with low family SES are read to less frequently on average, receive less language input, and have less access to resources for cognitive development, such as books in their home, or the ability to visit libraries or museums (Hart and Risley, 1995; Duncan and Brooks-Gunn, 1999; Bradley et al., 2001; Bradley and Corwyn, 2002; Evans, 2004). Relatedly, parental SES is negatively associated with child language abilities and executive functioning, including cognitive control (Noble et al., 2005, 2007; Hackman and Farah, 2009). Many of these effects of SES are mediated through differences in cognitive stimulation in the home (Linver et al., 2002; Sarsour et al., 2011; Hackman et al., 2015; Rosen et al., 2018). Children growing up in poverty and children who have experienced institutionalization show similar cognitive deficits, although institutionalization is associated with more profound behavioral differences. Thus, existing research provides initial support that poor development of language abilities and executive functions, including cognitive control, is related to exposure to deprivation.

### Threat

Within the DMAP, threatening experiences involve harm or threat of harm to the child or a close other. Experiences on the threat dimension include physical and sexual abuse, exposure to domestic violence, or direct exposure to community violence. The DMAP theory postulates that threatening experiences alter the neural circuitry underlying fear learning and emotional processing. This can produce disruptions in fear learning during development.

Within animal models, early-life exposure to stress has been associated with earlier development of fear learning and associated changes to brain function (Callaghan and Richardson, 2012, 2013). For example, pups maltreated by dams show avoidance of shock-associated odors earlier in development compared to standard-reared pups, suggesting earlier development of fear learning (Moriceau et al., 2009). Additionally, for rodents, regardless of maltreatment exposure, there is evidence that fear conditioning improves across age (Rudy, 1993; Britton et al., 2011).

Children show significant improvement in fear learning across childhood as well. Studies show evidence of increases in fear learning across age in early (Gao et al., 2010) and late childhood (Glenn et al., 2011; Jovanovic et al., 2014). While there are continuous improvements across age, there is less consistency across studies of when children can be said to have acquired fear conditioning. With regards to maltreatment, only one study in humans directly examines behavioral and physiological differences in fear acquisition and extinction associated with exposure to threat and deprivation in childhood (McLaughlin et al., 2016). In this study, adolescents with threat exposure involving abuse or domestic violence failed to discriminate between cues associated with threat (CS+) and those associated with safety (CS-), during the early phase of fear conditioning. No study has directly tested the possibility that exposure to maltreatment early in development will result in acquisition of fear learning at earlier ages, which would more directly replicate the work in rodents, cited above.

A large body of work has demonstrated that exposure to threatening experiences during childhood is associated with changes in emotion and physiological processing particularly in response to negative or aversive stimuli. Children exposed to abuse show heightened abilities to detect anger in facial expressions (Pollak and Kistler, 2002) and allocate more attention to angry faces (Shackman et al., 2007; Gibb et al., 2008; McCrory et al., 2010; Shackman and Pollak, 2014). In addition, children exposed to violence exhibit greater activation in the amygdala and broader salience network to negative relative to neutral cues (McCrory et al., 2011, 2013; McLaughlin et al., 2015). Finally, youth with abuse exposure exhibit overall blunted physiological responses to social threatening stimuli (Busso et al., 2017). Thus, exposure to violence in childhood appears to alter sensitivity to threat-related information in the environment by heightening awareness and enhancing neural responses to threat while simultaneously blunting physiological responses to these same stimuli.

### Current Study

Initial research demonstrates associations of deprivation and threat with cognitive control and fear learning when studied separately. In recent publications, we have examined these experiences together in the same model to investigate whether they have distinct associations with cognitive and emotional functioning. To date, this work has been carried out exclusively in adolescent samples. In these studies, we have observed that threat is associated with blunted physiological reactivity controlling for deprivation (Busso et al., 2017) as well as with difficulties with automatic emotion regulation problems (Lambert et al., 2017). In contrast, deprivation is associated with worse cognitive control and linguistic competence when controlling for exposure to threat (Lambert et al., 2017; Sheridan et al., 2017; Miller et al., 2018).

To date, no studies have examined the relative impact of experiences of deprivation and threat on functioning in early childhood. This represents a substantial gap in the literature for several reasons. First, experiences of adversity have sometimes been found to have stronger associations with psychopathology when they occur early in development (Manly et al., 2001; Kaplow and Widom, 2007; Dunn et al., 2013). Additionally, the brain develops rapidly in early childhood, producing dramatic associated changes in behavior (Casey et al., 2000). Finally, executive functions such as cognitive control – a primary outcome predicted to have differential associations with deprivation in the DMAP model – develops rapidly in early childhood (Anderson, 2002; Davidson et al., 2006; Best and Miller, 2010). Investigating the effects of deprivation and threat in early childhood has the potential to show how dimensions of experience impact the *development* of cognitive control and fear learning, as these processes are still in flux during early childhood.

The purpose of the current study was to evaluate how experiences of deprivation and threat are associated with behavior and physiology in early childhood (ages 4–7 years). Consistent with the DMAP theory and our previous work in adolescents, we expect that experiences of deprivation will be associated with worse cognitive control controlling for threatening experiences. In contrast, we anticipate that experiences of threat will be associated with poor physiological discrimination between threat and safety cues in a fear conditioning paradigm and blunted reactivity to the threat cues after controlling for depriving experiences. We additionally examined whether associations of deprivation and threat varied in their associations with cognitive control and fear learning across this early age range.

## Materials and Methods

### Participants

Sixty-four children aged 4–7 years old and a parent or legal guardian were recruited from a rural and suburban area in the southeast. Data was collected from as many participants as possible within a 1.5 year time period in which data collection was completed. Recruitment took place through a two-tiered approach. First, a general approach was used targeting families with low SES through listservs, craigslist, and other studies recruiting low SES populations. Then to ensure a diverse sample, families were recruited who were racial or ethnic minorities, had a primary caregiver who did not attend college, or met a clinical cut-off for concern on the Child Abuse Potential Inventory (CAPI) (166-point cut-off score) (Milner, 1994). Parents provided written consent in accordance with the Institutional Review Board. Children provided verbal assent between the ages of 4 and 6 years old and written assent if 7 years old. Exclusion criteria for participants included: (1) major medical conditions (e.g., HIV, cancer), (2) neurological illness (e.g., seizure disorders, migraines, multiple sclerosis), (3) factors limiting participant’s ability to complete proposed research (e.g., English fluency), and (4) pervasive developmental disorder (e.g., autism, Down’s syndrome). Children were not excluded for other diagnoses of psychopathology or psychological symptoms. Of these participants, one participant was excluded for inability to complete any behavioral tasks. Thus, the final sample included 63 children.

### Procedure

The present study was completed in one visit lasting approximately 3 h. Following informed consent procedures, parents of children in the study completed questionnaires assessing deprivation, threat, and symptoms of psychopathology. Children completed an IQ test, interviews about experiences of deprivation and experiences of threat, a behavioral task assessing cognitive control, and a fear conditioning and extinction paradigm.

### Measures

#### IQ

IQ was assessed through the Kaufman Brief Intelligence Test (KBIT-2) which is a brief IQ measure composed of verbal and non-verbal cognitive abilities for individuals 4–90 years old. The reliability of the composite IQ score from the KBIT-2 is 0.93 in a normative sample (Bain and Jaspers, 2010).

### Deprivation

A deprivation score was derived from multiple measures. Each measure was standardized to create a z-score. These z-scores were summed to create a total deprivation score. The data from the HSQ and parental education was transformed by subtracting the total score for each child from the maximum score possible on each measure. As a result, for all measures, higher totals indicated a higher level of deprivation.

#### Neglect

Neglect was assessed through the Multidimensional Neglectful Behavior Scale (MNBS-CR), a child interview measure (Kantor et al., 2004). The MNBS-CR is an interview for young children to assess neglect using cartoon-based items tailored to the participant’s gender and the gender of their primary caregiver. The reliability of the neglect items ranges from 0.66 to 0.94 depending on the sample (Kantor et al., 2004). The present study assessed neglect using the emotional neglect, cognitive neglect, physical neglect, supervisory neglect, and abandonment items (43 items total; alpha = 0.71).

#### Cognitive Stimulation

The level of age-appropriate scaffolded learning opportunities provided to the child was assessed using the Home Screening Questionnaire (HSQ) (Frankenburg and Coons, 1986). The HSQ is a parent-report measure based on the Home Observation for the Measurement of the Environment (HOME), an observational measure for use in children’s homes to assess cognitive stimulation and emotional support in the home (Bradley et al., 2003). The HOME and the HSQ identified the same children in need of support 86% of the time, suggesting that the HSQ may be used if home observation is not possible. The HSQ has good test-retest reliability in children above 3 years old (0.86) (Frankenburg and Coons, 1986). The current study utilized the sum of the HSQ with five items removed that assessed spanking and parental decision-making in the household, which were not conceptualized as a part of cognitive stimulation (56 items in original scale, 51 items utilized).

#### Parental Education

Parental education was assessed using the Macarthur Scale of Subjective Social Status (Adler and Stewart, 2007). Parental education was measured as the average of educational attainment for both primary caregivers of the child. If there was one primary caregiver, then the parental education of the one primary caregiver was utilized. Possible responses ranged from “Less than high school diploma (1)” to “Professional degree (5).”

### Threat

The threat dimension was also comprised of a score derived from multiple measures that were standardized and summed to create a total threat score.

#### Violence Exposure

Exposure to violence was assessed using the Violence Exposure Scale for Children-Revised (VEX-R), a child interview measure (Fox and Leavitt, 1995). The VEX-R is a 21-item, cartoon-based interview used to assess young children’s exposure to abuse, domestic violence and community violence. VEX-R has good internal consistency ranging from 0.80 to 0.86 (Shahinfar et al., 2000; Kolko et al., 2010). The current study utilized the sum of the total items in which children reported on exposure to violence perpetrated by a teenager or an adult (Cronbach’s alpha = 0.81).

#### Partner Violence

Presence of domestic violence in the home was assessed using the Conflict Tactic Scales (CTS-2), a parent-report measure (Straus et al., 1996). The CTS-2 consists of 39 items in five subscales: physical assault (12 items), psychological aggression (8 items), negotiation (6 items), injury (6 items) and sexual coercion (7 items). All subscales have good internal consistency (0.79–0.95) (Straus et al., 1996). The total sum of the physical assault, psychological aggression, injury, and sexual coercion subscales was used in the present study (alpha = 0.94).

#### Physical Abuse

The likelihood of physical abuse was assessed using the CAPI, a parent-report measure (Milner et al., 1988). The CAPI is a 160-item scale which screens for parental attitudes which indicate high risk for present or future physical abuse. Internal consistency with different populations ranges from 0.84 to 0.94 (Milner et al., 1988). In the present sample, alpha = 0.90.

### Cognitive Control

**Figure 2:**
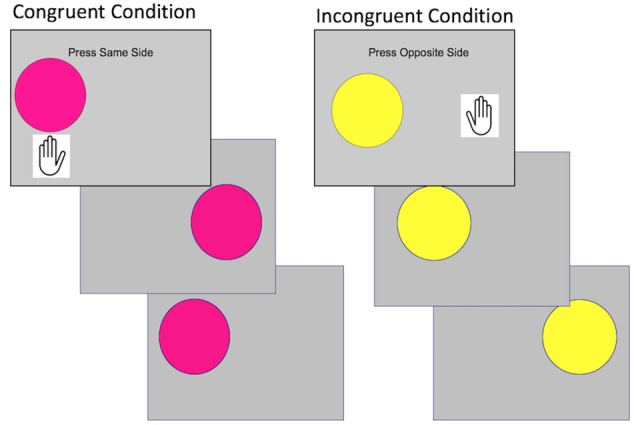
Illustration of the cognitive control task showing the congruent condition in which the child presses on the same side and the incongruent condition in which the child presses on the opposite side. Within the block design, each image is presented for 2 s in a block of five trials.

The Simon task is a cognitive control task frequently used in children (Kharitonova et al., 2013). In this task (Figure 2), children press a button on the same side of a screen if the stimulus is one color (congruent trials), and the opposite side from the stimulus if the stimulus is a different color (incongruent trials). On incongruent trials, children must inhibit a prepotent response (to press on the same side) in favor of a conflicting response (to press on the opposite side). This task has been used with children 5–10 years old (Kharitonova et al., 2013). Cognitive control is reflected in the difference between performance on incongruent relative to congruent trials. Behavioral performance was quantified through inverse efficiency, a measure that incorporates both speed and accuracy into one metric. Inverse efficiency is commonly used on tasks that demonstrate a speed-accuracy tradeoff (Townsend and Ashby, 1978). Inverse efficiency is calculated by dividing mean reaction time of correct responses by the proportion of correct responses (Bruyer and Brysbaert, 2011).

Three children were unable to complete the task due to poor comprehension or behavioral difficulties. Participants were considered outliers if their task performance exceeded two standard deviations from the group means, resulting in 58 children with valid cognitive control data.

### Fear Conditioning

**Figure 3:**
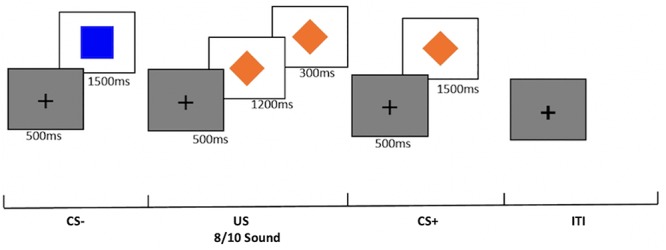
Illustration of the block-design fear acquisition and fear extinction task. In the first block, children are presented with a set of 10 CS- trials of the above duration, followed by 10 US trials, 10 CS+ trials, and ITI of the same duration. In 8/10 US trials, children hear a loud aversive sound. Following the first block, block order is randomized for each set of trials (CS-, US, CS+, ITI). Children are presented with four blocks of each trial type in fear acquisition. In fear extinction, children are presented with four blocks of three trial types (CS-, CS+, ITI).

The present study used a block design fear conditioning and extinction paradigm designed for use with young children (Figure 3). In contrast to more common “event related” fear conditioning paradigm, this task was designed to maximize the likelihood that young children would acquire fear conditioning. This was done by showing separate blocks of reinforced threat cues (US), non-reinforced threat cues (CS+), and safety cues unassociated with aversive reinforcement (CS-). This type of blocked fear conditioning task has been previously used with children and produces successful discrimination of threat and safety cues (Jovanovic et al., 2014; Silvers et al., 2016; van Rooij et al., 2017). In the task, two shapes (blue square and orange diamond) were used as conditioned stimuli, randomized across participants. Children viewed 12 stimulus blocks during acquisition: 4 blocks of the CS+ reinforced with an aversive loud sound (US); 4 blocks of the CS+ non-reinforced without the US (CS+), and 4 blocks of the CS-. In each block, children viewed 10 stimuli. The reinforcement rate was 80% in the US blocks. Before and after fear acquisition, children reported which shape was on the screen when they heard a sound. During extinction, children viewed 8 stimulus blocks: 4 blocks of the CS+ and 4 blocks of the CS-. On 2/10 of the trials in all blocks, children pressed to a dot on top of the shape to ensure attention during the task.

Questionnaire data and skin conductance data were collected. Fear learning is measured by the amplitude of skin conductance responses (SCRs) during acquisition. Children were given a forced-choice question to distinguish between the CS+ and the CS- after fear acquisition and fear extinction asking, “Which shape was on the screen when you heard a sound?” Physiological data was collected continuously using Mindware. Skin conductance was measured through two electrodes filled with sodium chloride gel attached to the palm of the non-dominant hand. Data are sampled at 1000 Hz. Data were filtered and smoothed using Mindware. SCRs were calculated following standard procedures as the difference in the 1–5 s following stimulus onset, with a minimum response of 0.05 microsiemens (μs) (Braithwaite et al., 2013). All data was manually examined to verify that all peaks in skin conductance after an event were identified for each participant, non-responders (*N* = 3) were excluded from further analysis.

Using Mindware data analysis software, the amplitude of SCRs was collected for each trial type (US, CS+, and CS-) across acquisition and extinction blocks. A range-correction on the amplitude of skin conductance was done to correct for inter-individual variance (i.e., some children showed large variability in SCRs, while others showed little variability). This range correction was done by dividing each skin conductance amplitude value by the maximum skin conductance value for each participant (Lykken and Venables, 1971; Boucsein, 2012). If the range-correction of the data is conducted with a square root transformation, the results of the study are substantively unchanged.

Ordinary least squares multiple regression models were used to predict amplitude of SCR to the CS+ controlling for amplitude to the CS- to assess associations with fear acquisition. The four blocks of fear acquisition were divided into early fear acquisition (Blocks 1–2) and late fear acquisition (Blocks 3–4) based on prior literature (Jovanovic et al., 2014; McLaughlin et al., 2016).

Five children were unable to complete the task. Out of these five children, two children did not provide consent, one child was allergic to the materials, and two children were unable to complete the task due to behavioral issues. Due to technical problems, data from six children was unusable. Additionally, six children aborted the task during fear acquisition or fear extinction. Thus, 46 children were included in skin conductance analyses with complete data.

### Statistical Analyses

Ordinary least squares multiple regression models were used to predict task performance on the cognitive control task. For the cognitive control task, the main effects were examined to establish behavioral differences between congruent and incongruent trials. Next, the hypothesized model was constructed in which the dependent variable was inverse efficiency on incongruent trials. Predictors included inverse efficiency on congruent trials, age, gender, IQ, threat score, and deprivation score. Next an age × deprivation interaction term and an age × threat interaction term were separately included.

Separate linear regression models were conducted for early and late fear acquisition. Predictors included amplitude of SCR to the CS-, age, gender, IQ, threat score, and deprivation score. Subsequently, age × threat and age × deprivation interactions were separately examined. In a separate analysis, reactivity to the US was examined using a multiple regression model with the same set of predictors. This model was designed to assess associations with overall physiological reactivity.

## Results

### Sample Characteristics

Children ranged from 4 to 7 years of age (*M* = 74.1 months, *SD* = 14.1). The IQ of the sample represented a normal distribution of IQ with a mean of 99.6 and standard deviation of 15. The dimensional measures of deprivation and threat were constructed with a mean score of 0 from the sum of the z-scores of relevant measures (Deprivation *M* = 0.0, *SD* = 2.2, Threat *M* = 0.0, *SD* = 2.3). 36 children identified as female (56.3%) and 27 children identified as male (42.2%). Sample characteristics are presented in Table A1. Age, gender, and IQ were used as covariates in all analyses.

### Deprivation and Threat Correlations

Parental education was significantly correlated with the HSQ (*r* = 0.36, *p* < 0.01), which assesses cognitive stimulation in the home, and the MNBS-CR (*r* = -0.27, *p* < 0.05), which assesses child-reported neglect. The HSQ and MNBS-CR were not significantly correlated (*r* = -0.19, *p* = ns). All measures of threat were significantly correlated with each other (*r* > 0.35, *p* < 0.01 for all measures). Finally, deprivation and threat were not correlated in this sample (*r* = 0.15, *p* = 0.23).

### Cognitive Control Main Effects

Children responded with higher accuracy on congruent trials than incongruent trials (86.6% on congruent vs. 81.5% on incongruent; *t* = 3.75, *p* < 0.001). Children responded faster on congruent (697.83 ms) vs. incongruent (745.30 ms) trials (*t* = -4.79, *p* < 0.001). Children also performed better on congruent trials than incongruent trials using the inverse efficiency measure (*t* = -6.35, *p* < 0.001).

### Cognitive Control and Experience

**Figure 4:**
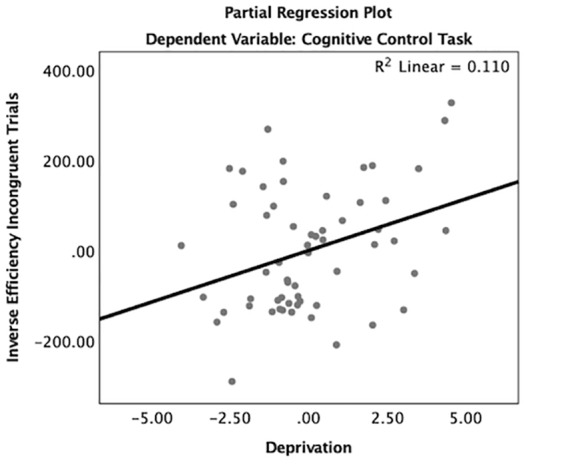
Partial regression plot of deprivation on cognitive control task performance. There was a significant association between deprivation and worse cognitive control task performance on incongruent trials controlling for congruent trials, age, gender, IQ, and threatening experiences.

Consistent with hypotheses, deprivation was significantly associated with inverse efficiency of incongruent trials when controlling for inverse efficiency of congruent trials, age, gender, IQ, and threatening experiences (β = 0.14, *p* < 0.05, Table A2 and Figure 4). Children with higher levels of deprivation exhibited worse cognitive control performance. In contrast, threatening experiences were not associated with task performance. Age did not interact with deprivation (β = -0.07, *p* = 0.84) or threat (β = 0.30, *p* = 0.44) to predict performance on the cognitive control task.

### Fear Conditioning Main Effects

Following fear acquisition, 87% of children reported contingency awareness of the US-CS+ association, and 82% had contingency awareness following fear extinction. Children responded with 83.9% accuracy to a cue used to monitor attention to the task during fear acquisition and 84.8% accuracy during fear extinction. In early and late fear acquisition, there were no significant main effects of the amplitude of skin conductance response comparing the CS+ and CS- (early *t* = -1.39, *p* = 0.17; late *t* = 0.02, *p* = 0.98). There were significant differences between the US compared to the CS+ (early *t* = -3.27, *p* < 0.01; late *t* = -2.95, *p* < 0.01) and the US compared to the CS- (early *t* = -4.40, *p* < 0.01; late *t* = -2.80, *p* < 0.01) across both early and late fear acquisition.

### Acquisition of Fear Conditioning and Experience

**Figure 5:**
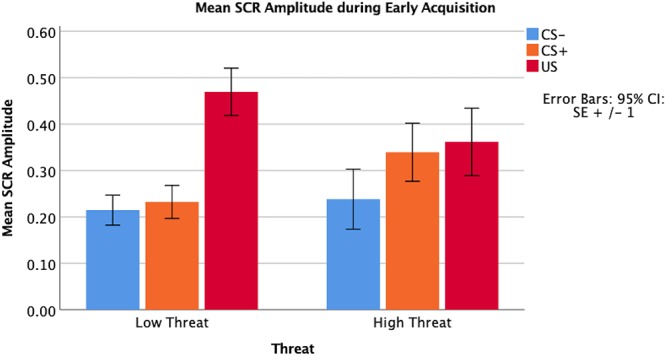
Amplitude of SCR main effects by threat exposure during early fear acquisition.

**Figure 6:**
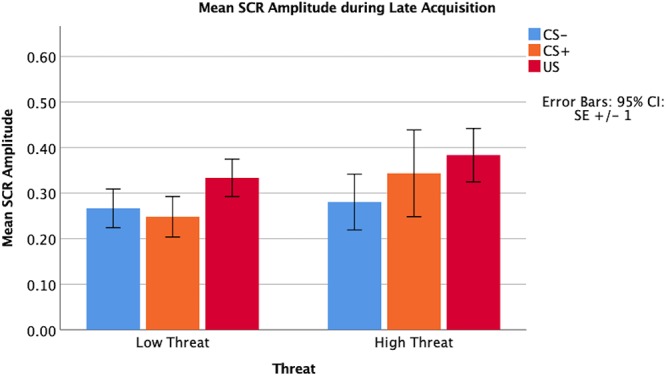
Amplitude of SCR main effects by threat exposure during late fear acquisition.

**Figure 7:**
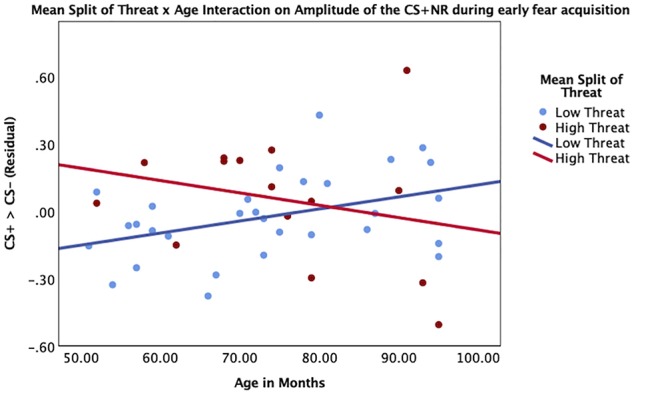
Interaction between age and threat on amplitude of the CS+ during early fear acquisition. Threat exposure significantly interacted with age to predict amplitude of SCR to the CS+ during early fear acquisition controlling for age, gender, IQ, and deprivation. At lower levels of threat, age was associated with increases in physiological differentiation between the CS+ and the CS-. At higher levels of threat, age was unrelated to SCR amplitude to the CS+.

Threat was not significantly associated with amplitude of the SCR to the CS+ during early (β = 0.07, *p* = 0.67) or late (β = -0.01, *p* = 0.99) fear acquisition, nor was deprivation (early β = 0.11, *p* = 0.53; late β = 0.08, *p* = 0.96) (Figure 5, 6). Deprivation did not interact with age to predict SCR to the CS+ (early β = 0.22, *p* = 0.83; late β = -1.43, *p* = 0.12). However, threat exposure interacted with age to predict SCR to the CS+ during early fear acquisition (β = -2.43, *p* = 0.02) but not late acquisition (β = -0.08, *p* = 0.94) (Table A3). To probe this interaction, the conditional effects of threat were examined at the mean of threat and one standard deviation above and below the mean. At low levels of threat, age was positively associated with amplitude of the SCR to the CS+ (*t* = 2.72, *p* < 0.05). That is, as age increased, physiological differentiation between the CS+ and CS- increased. At average and high levels of threat, age was unrelated to SCR amplitude to the CS+ (mean: *t* = 0.79 *p* = 0.43, 1 SD above: *t* = -1.65 *p* = 0.11), meaning that children exposed to threat exhibited SCR responses to the CS+ across the entire age range of our sample (Figure 7).

### Physiological Reactivity and Experience

**Figure 8:**
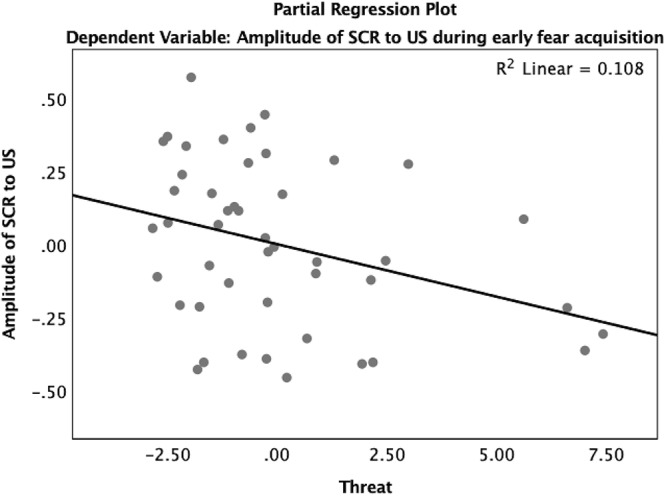
Partial regression plot of threat on amplitude of SCR to the US during early fear acquisition. There was a significant association between threat and reduced reactivity to the US measured by the amplitude of SCR during early fear acquisition controlling for age, gender, IQ, and deprivation.

Reactivity to the US was analyzed using a multiple regression model predicting amplitude of SCR to the US. In early acquisition, threat was significantly associated with the amplitude of the SCR to the US (β = -0.36, *p* < 0.05) where higher levels of threat were associated with reduced SCR amplitude (Table A4 and Figure 8). In late acquisition, threat was not associated with SCR amplitude to the US (β = -0.05, *p* = 0.72), nor were other covariates. Age x threat and age x deprivation interactions were added to these models separately. The interaction between age and threat did not significantly predict amplitude of the SCR to the US in early (β = -1.01, *p* = 0.42) or late (β = 0.30, *p* = 0.81) acquisition nor did the interaction between age and deprivation (early β = -1.61, *p* = 0.13; late β = -0.90, *p* = 0.39).

## Discussion

In the present study, we examined associations of deprivation and threat with cognitive control and fear learning in early childhood. Consistent with hypotheses, children with higher levels of deprivation exhibited worse performance on incongruent trials of the cognitive control task controlling for congruent trials, age, gender, IQ and threatening experiences. Thus, children with more experiences of deprivation had more difficulty inhibiting a prepotent response in favor of another response. Additionally, threatening experiences interacted with age to predict fear learning measured by physiological response. Differential physiological response associated with fear learning emerged at older ages for children exposed to low levels of threat. For children with higher levels of threat exposure, there was evidence of differential skin conductance response across all ages. Across tasks, differential outcomes were observed associated with deprivation and threat.

We hypothesized that exposure to deprivation would be associated with impaired cognitive control after controlling for threatening experiences. Prior research has found that children raised in institutions, a severe experience of deprivation, exhibit poor performance on cognitive control tasks (Tibu et al., 2016). Similarly, young children from families with low SES have difficulties with cognitive control (Noble et al., 2005; Sarsour et al., 2011; Lawson et al., 2018). Therefore, the findings of the present study are consistent with an overall body of work that suggests deprivation in early childhood is associated with impaired cognitive control. Finally, prior work examining the deprivation and threat model has also found evidence that deprivation was associated with impaired cognitive control and other forms of executive function when measured during adolescence. Poverty was associated with impaired inhibitory control and working memory in adolescence controlling for child abuse and exposure to community violence (Lambert et al., 2017; Sheridan et al., 2017; Rosen et al., 2018). The present study replicates and extends this work by demonstrating that these associations are observable in early childhood and continue to be robust when deprivation is measured using a more multi-faceted and comprehensive index, not a single indicator. In sum a growing body of evidence supports that deprivation specifically is associated with impaired inhibitory control relative to other experiences of adversity. Furthermore, the current study suggests that the specific association between deprivation and inhibitory control begins in early childhood, highlighting a need to intervene on children experiencing deprivation to address this specific and stable deficit.

We additionally hypothesized that threat would be associated with difficulty discriminating between threat and safety cues during fear conditioning. One prior study examined the impact of threatening experiences on fear learning in adolescence (McLaughlin et al., 2016). In this study, adolescents with high levels of threat exposure did not differentiate between the CS+ and CS- in terms of SCRs. In the present study, questionnaire data indicate that children in this age range were broadly able to attend to the task and later could identify which cue occurred in conjunction with an unpleasant sound. In physiological measures, we observed that relative to those exposed to low levels of threat, young children 4–5 years old with high levels of threatening experience demonstrated *greater* differentiation between the threat and safety cue during early acquisition. For children with lower levels of threat, differentiation between the CS+ and the CS- improved with age, which is consistent with other findings that older children are better able to differentiate between the CS+ and the CS- (Glenn et al., 2011; Jovanovic et al., 2014). This data suggests that having more threatening experiences in early childhood is associated with the ability to distinguish CS cues in fear learning earlier. No associations were observed between deprivation and fear learning.

Our observation of earlier ability to distinguish between fear stimuli for children exposed to threat is consistent with a body of animal literature suggesting that threatening experiences are associated with an accelerated developmental trajectory of fear learning and associated changes to brain function (Tottenham, 2009, 2012a). In rodent studies, standard-reared pups have been compared to pups placed in an environment with reduced nesting material, leading to pup maltreatment by the dam (Callaghan and Tottenham, 2016). Pups raised with reduced nesting material show an accelerated developmental trajectory of avoidance of shock-associated odors compared to standard-reared pups, suggesting earlier development of fear learning (Moriceau et al., 2009). Furthermore, these pups show greater avoidance of shock-associated odor earlier, suggesting earlier development of neural regions required in fear conditioning processes (Callaghan and Tottenham, 2016). Overall, findings from the present study support theoretical models that adversity in early childhood may be associated with accelerated development of fear learning behavior potentially through associated changes to brain structure and function (Tottenham, 2009, 2012a; Callaghan and Tottenham, 2016). It may be that this acceleration is specifically observable in early childhood. By the time children are adolescents, youth in unsafe environments have had many experiences of threat that occur unpredictably, which may be associated with a subsequent lack of differentiation in physiological responding between the CS+ and CS- (McLaughlin et al., 2016). Future work taking a longitudinal approach will be invaluable in identifying the trajectory of the development of fear learning for children with and without early threatening experiences.

Children with threat exposure also showed significantly blunted reactivity to the US across time regardless of age. Numerous other studies have shown blunted SCRs to threatening stimuli in childhood and adolescents across studies and paradigms (MacMillan et al., 2009; Ouellet-Morin et al., 2011; Trickett et al., 2014; Busso et al., 2017). In addition, in adults, threatening experiences across the lifespan have been associated with blunted reactivity as measured by startle response, heart rate, cardiac output, and skin conductance in the context of fearful or threatening experiences (Lovallo et al., 2012; McTeague and Lang, 2012; D’Andrea et al., 2013; Heleniak et al., 2016). Thus, the present data are entirely consistent with a large body of work showing that individuals exposed to more threatening experiences have overall blunted reactivity. The current study extends prior findings to provide preliminary evidence that this blunted reactivity is observable even in early childhood.

Taken together, these findings support the specificity of deprivation in its association with cognitive control deficits and the specificity of threat in its association with the development of fear learning. The findings demonstrate the importance of measuring and considering the impacts of deprivation and threat separately in studies examining early adversity. Prior work on early adversity has typically measured all types of early adversity together, most commonly as a cumulative risk measure of the total number of experiences of early adversity (e.g., Evans et al., 2013). Given the specificity of deprivation and threat in their associations with behavior in early childhood, combining all measures of early adversity together may mask specific associations between dimensions of experience and behavior. This runs the risk of both underestimating true effects, and, most importantly, decreasing the likelihood that specific pathways will be identified stunting the development of novel preventive interventions.

### Study Limitations and Future Directions

No prior studies have explored the impact of the deprivation dimension of experience and the threat dimension of experience on behavior in early childhood. The study makes a novel contribution by examining early adversity as dimensions of experience in early childhood when the brain is rapidly developing, and by controlling for the other dimension of experience in all analyses. However, several limitations should be noted. First, these findings come from a small sample drawn from a single geographic location. Therefore, it is unknown how these findings would apply to samples acquired across diverse geographical areas. Second, age, gender, IQ, and the other dimension of experience (either deprivation or threat), were used as covariates in all analyses. In larger samples, there are additional covariates that would be recommended to account for other differences in children who have experienced early adversity. These could include measures of prenatal exposure to illegal substances, prenatal maternal stress, nutrition, lead exposure, and other environmental toxic exposures. Third, this study was cross-sectional, and the results of the present study are correlational: experiences of deprivation and threat were not manipulated. For that reason, strong causal arguments are not possible within this model. Finally, index scores of deprivation and threat were utilized due to the relatively small sample size of the current study. In a larger sample, measuring deprivation and threat as latent constructs from multiple measures may be possible.

In the present study, we extended prior work by demonstrating specific effects of deprivation and threat on behavior in early childhood while controlling for the other dimension of experience. These findings provide additional support for conceptual models arguing against a one-size-fits-all approach to ELA (McLaughlin et al., 2014; Sheridan and McLaughlin, 2014) and suggest instead that the developmental consequences of different types of adversity are at least partially distinct. Future work should investigate the relationship between deprivation and threat in its influence on brain structure and function in early childhood in order to examine evidence for neural mechanisms linking dimensions of experience and alterations in behavior in early childhood.

## Ethics Statement

Parents provided written consent. Children provided verbal assent between the ages of 4 and 6 years old and written assent if 7 years old. Exclusion criteria for participants included: (1) major medical conditions (e.g., HIV, cancer), (2) neurological illness (e.g., seizure disorders, migraines, multiple sclerosis), (3) factors limiting participant’s ability to complete proposed research (e.g., English fluency), and (4) pervasive developmental disorder (e.g., autism, Down’s syndrome). Children were not excluded for other diagnoses of psychopathology or psychological symptoms. Of these participants, one participant was excluded for inability to complete any behavioral tasks. Thus, the final sample included 63 children. All procedures were approved by the Institutional Review Board at the University of North Carolina, Chapel Hill.

## Author Contributions

LM, MS, and KM contributed to conception and design of the study and contributed to interpretation of data. LM and JS conducted data collection. LM, JS, MS, and AM contributed to statistical analysis. LM wrote the first draft of the manuscript. All authors contributed to manuscript revision, read, and approved the submitted version. Study was supervised by MS.

## Conflict of Interest Statement

The authors declare that the research was conducted in the absence of any commercial or financial relationships that could be construed as a potential conflict of interest.

## Figures and Tables

**Table A1:** Distribution of sample characteristics (*N* = 63).

	Minimum	Maximum	Mean	*SD*
Age (months)	51	95	74.14	14.06
IQ	61	131	99.60	15.27
Parent education^a^	1	5	2.49	2.38
Deprivation	-3.9	6.1	0.0	2.2
Threat	-2.6	7.6	0.0	2.3
	**%**	**n**		
Female	56.3	36		
**Race/Ethnicity**		
White	44.4	28		
African American	34.9	22		
Hispanic/Latino	9.5	6		
Asian	1.6	1		
Multiracial/Other	9.5	6		
CAPI Cutoff^b^	26.6	17		
CTS-2 Cutoff^c^	26.6	17		
VEX-R Cutoff^d^	21.9	14		

^a^*Average of parent education if child had two caregivers or primary caregiver education (Less than high school diploma = 1 to Professional degree = 5).*^b^*Child Abuse Potential Inventory Cutoff is a score of above 166.*^c^*Conflict Tactics Scale (CTS-2) Cutoff is measured as endorsing an item in the Physical Abuse, Injury, and Sexual Coercion subscales.*^d^*VEX-R Cutoff is measured by endorsing critical items (e.g., being pushed or shoved really hard by an adult, being slapped really hard by an adult, being beaten up by adult).*

**Table A2:** Multiple regression analysis of cognitive control task (*N* = 58).

Variables	*B*	*SE B*	β	*T*	*p*
Intercept	731.57	263.10		2.78	0.008
Inverse efficiency of congruent trials of cognitive control task	1.08	0.11	0.75	9.82	<0.001
Age	-4.74	1.87	-0.19	-2.54	0.014
Gender	48.98	38.16	0.07	1.28	0.205
IQ	-3.01	1.45	-0.12	-2.08	0.043
Deprivation	22.79	9.06	0.14	2.52	0.015
Threat	-5.29	8.03	-0.04	-0.66	0.513

**Table A3:** Multiple regression analysis of amplitude of SCR to the CS+ during early fear acquisition (*N* = 46).

Variables	*B*	*SE B*	β	*t*	*p*
Intercept	-0.37	0.26		-1.41	0.167
Amplitude of SCR to the CS- during early fear acquisition	0.22	0.16	0.21	1.34	0.189
Age	0.00	0.00	0.25	1.71	0.095
Gender	0.08	0.07	0.18	1.12	0.269
IQ	0.00	0.00	0.17	1.07	0.292
Deprivation	0.01	0.02	0.12	0.76	0.453
Threat	0.21	0.09	2.47	2.41	0.021
Interaction of age and threat	-0.00	0.00	-2.43	-2.36	0.023

**Table A4:** Multiple regression analysis of amplitude of SCR to the US during early fear acquisition (*N* = 46).

Variables	*B*	*SE B*	β	*T*	*p*
Intercept	0.84	0.36		2.33	0.025
Age	0.00	0.00	-0.01	0.09	0.929
Gender	-0.06	0.09	-0.11	-0.71	0.483
IQ	-0.00	0.00	-0.17	-1.01	0.319
Deprivation	0.00	0.02	0.00	0.02	0.984
Threat	-0.04	0.02	-0.33	-2.21	0.033
